# Observation of time-dependent psychophysical functions and accounting for threshold drifts

**DOI:** 10.3758/s13414-015-0865-x

**Published:** 2015-03-26

**Authors:** Robert J. Doll, Peter H. Veltink, Jan R. Buitenweg

**Affiliations:** Biomedical Signals and Systems, MIRA Institute for Biomedical Technology and Technical Medicine, University of Twente, Zuidhorst, ZH222, Drienerlolaan 5, P.O. Box 217, 7500AE Enschede, The Netherlands

**Keywords:** Non-stationarity, Threshold, Slope, Tracking, Simulations, Psychometrics

## Abstract

Methods to obtain estimates of psychophysical functions are used in numerous fields, such as audiology, vision, and pain. Neurophysiological and psychological processes underlying this function are assumed to remain stationary throughout a psychophysical experiment. However, violation of this assumption (e.g., due to habituation or changing decisional factors) likely affects the estimates of psychophysical parameters. We used computer simulations to study how non-stationary processes, resulting in a time-dependent psychophysical function, affect threshold and slope estimates. Moreover, we propose methods to improve the estimation quality when stationarity is violated. A psychophysical detection experiment was modeled as a stochastic process ruled by a logistic psychophysical function. The threshold was modeled to drift over time and was defined as either a linear or nonlinear function. Threshold and slope estimates were obtained by using three estimation procedures: a static procedure assuming stationarity, a relaxed procedure accounting for linear effects of time, and a threshold tracking paradigm. For illustrative purposes, data acquired from two human subjects were used to estimate their thresholds and slopes using all estimation procedures. Threshold estimates obtained by all estimations procedures were similar to the mean true threshold. However, due to threshold drift, the slope was underestimated by the static procedure. The relaxed procedure only underestimated the slope when the threshold drifted nonlinearly over time. The tracking paradigm performed best and therefore, we recommend using the tracking paradigm in human psychophysical detection experiments to obtain estimates of the threshold and slope and to identify the mode of non-stationarity.

## Introduction

Methods to obtain estimates of psychophysical functions describing processes of underlying sensory mechanisms are used in many fields of study (e.g., hearing, vision, or tactile studies). The psychophysical function describes the probability that a stimulus is detected given the stimulus strength and has a sigmoidal form (Gold & Ding, 2013; Leek, Hanna, & Marshall, 1991). Psychophysical parameters describing the psychophysical function, such as the threshold and the slope, can serve as a quantifier of disease, as in studies of audiology (McFadden, 1983), vision (Chauhan, Tompkins, LeBlanc, & McCormick, 1993; Wallis, Baker, Meese, & Georgeson, 2013), and pain (Sandkühler, 2009).

In a simple stimulus detection experiment, subjects are presented several stimuli with varying amplitudes to which the corresponding responses (i.e., detected or not detected) are recorded. From these stimulus-response pairs (SRPs), an estimate of the psychophysical function can be obtained (see Kingdom and Prins (2009) for an introduction to psychophysical methods). The threshold parameter is a commonly used measure to describe this function and is defined as the amplitude resulting in a 50% detection probability (Klein, 2001; Treutwein, 1995). The steepness of the psychophysical function at the threshold level is described by the slope parameter and provides information about the reliability of stimulus detection by the subject (Gold & Ding, 2013; Strasburger, 2001).

In practice, the neurological and psychological processes underlying the psychophysical function are assumed to remain stationary throughout estimations of threshold and slope in psychophysical experiments. However, fatigue, loss of attention, or a change in decisional criteria (Fründ, Haenel, & Wichmann, 2011; Leek et al., 1991) can result in violation of this assumption. As a result, estimates of the psychophysical threshold and slope might be impaired and are possibly unreliable.

**Fig. 1:**
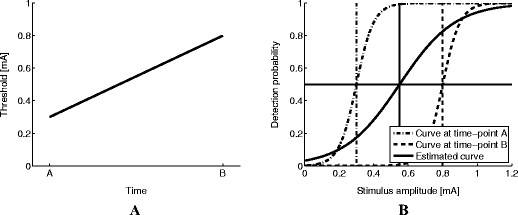
(**a**) True thresholds can drift over time resulting in a different threshold value at time-point B than at time-point A. (**b**) A drift over time in the threshold can be represented as a shift of the psychophysical function to the right (e.g., from the dash-dotted curve at time-point A to the dashed curve at time-point B). A curve similar to the solid line will be estimated when stationarity of the threshold is assumed

A conceivable consequence of violation of the stationarity assumption is demonstrated in Fig. 1. Figure 1a presents how a threshold can drift within an experiment from time-point A to B, resulting in a rightward shift of the psychophysical function on the stimulus axis. This rightward shift is depicted in Fig. 1b. The two dashed lines depict the true psychophysical function at time-points A and B, respectively. In between the two time-points, the function shifts as a linear function from time-point A to B while the slope parameter remains stationary. The solid black line represents the estimate of the psychophysical function from stimulus response pairs obtained between time-points A and B when stationarity would have been assumed. As all stimulus response pairs contribute equally to the estimation, the estimate of the threshold will be similar to the averaged true threshold over the time interval A-B. As a result, the slope of the estimated curve is underestimated and therefore falsely suggests a lower accuracy of the subject to discriminate between stimulus intensities.

A practical example of where non-stationarities are known to occur is when estimating the nociceptive function. Changes in nociceptive thresholds can occur, for example, due to clinical interventions (Olesen et al., 2013) and experimental conditioning stimuli (Pud, Granovsky, & Yarnitsky, 2009). Moreover, nociceptive detection thresholds can show a continuous increase over time during a 10-minute experiment (Doll, Buitenweg, Meijer, & Veltink, 2014). Additionally, observation of the effect of time on estimations could be a relevant indicator of disease (e.g., reduced habituation is present in fibromyalgia and migraine patients (Smith et al., 2008; Valeriani et al., 2003)).

If unwanted, the effect of time on the psychophysical function can be minimized by carefully preparing the experiment, such as increasing the time between two consecutive stimuli (von Dincklage, Olbrich, Baars, & Rehberg, 2013). However, these preparations could be impractical for clinical purposes, where only a limited amount of time is available for psychophysical recordings. For these purposes, it is important to be aware of the possibility that the stationarity assumption can be violated. Therefore, the purpose of this study was to observe how a non-stationary process, resulting in a time-dependent psychophysical function, can be identified and how this affects threshold and slope estimates. Moreover, we study how these estimates can be improved by two different estimation procedures.

As a first strategy to improve estimations of the psychophysical functions, the assumption of a stationary threshold could be relaxed by allowing time to have a linear effect on the threshold. Allowing this can be done by including the times at which stimuli were presented in the estimation process (e.g., include stimulation time as a covariate in (generalized) linear regression models). Doing so might improve the slope estimates. However, this improvement might depend on the type of non-stationarity of the underlying processes. Not only linear drifts of the psychophysical function but also more complex nonlinear drifts might occur (Milne, Kay, & Irwin, 1991).

As a second strategy, nonlinear changes in a threshold over time can be observed by using a threshold tracking paradigm. This paradigm uses a time-window including a number of the most recent SRPs to obtain a momentary estimate of the threshold (Doll et al., 2014; von Dincklage, Hackbarth, Schneider, Baars, & Rehberg, 2009). This window is shifted each time a new single SRP is obtained. Therefore, this paradigm could be used as a first identifier of non-stationary behavior.

How threshold and slope estimates are affected by non-stationary processes depends on its time-dependent properties and on the estimation procedure. In human psychophysical experiments, the true threshold and slope are unknown and thus can only be estimated. However, computer simulations allow the true psychophysical parameters to be defined and therefore allow evaluating the estimates. We used a Monte Carlo simulation approach to generate stimulus response pairs. A psychophysical function underlying a non-stationary process was modeled by means of a drifting threshold. The true threshold was defined as (1) a constant function, (2) a linear increasing function, or (3) a saturating exponential function. Three different estimation procedures were used to obtain estimates of the threshold and the slope of the psychophysical function using the simulated SRPs. In addition to the simulations, we illustrate the estimation procedures on data of two human subjects coming from a previous study (Doll et al., 2014).

## Methods

Estimates of the psychophysical functions, in terms of thresholds and slopes coming from three different estimation procedures, were compared by means of Monte Carlo simulations. The simulation procedures described below are similar to the procedures described in Doll et al. (2014). Several realizations of a psychophysical experiment were simulated using a stochastic psychophysical function to simulate the responses to stimulus amplitudes. Threshold drift was included in the simulations to model a non-stationary underlying process. The true threshold was defined as (1) a constant function, (2) a linear increasing function, and (3) a saturating exponential function with two different time constants. Thresholds and slopes were estimated using one out of the three estimation procedures described in the section “Threshold and slope estimation” below. For each of the 12 experiments presented in this paper, 10,000 realizations were simulated. In addition to the simulations, psychophysical data coming from two human subjects were used to illustrate the different estimation procedures in practice. All simulations and analysis were performed with MATLAB 8.1. (MathWorks, Inc., Natick, MA).

## Psychophysical model and model parameters

**Fig. 2:**
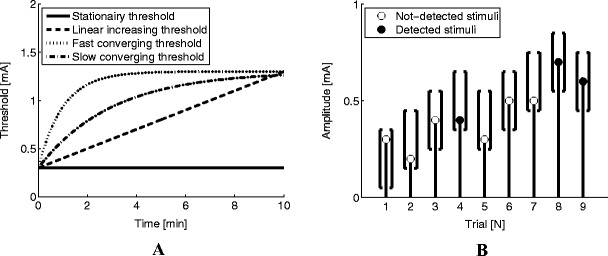
(**a**) Four threshold functions were used: a constant threshold of 0.3 [mA], a linear drifting threshold with a drifting rate of 0.1 [(mA)/min], a slow converging threshold with a time-constant of 3 [min], and a fast converging threshold with a time-constant of 1 [min]. (**b**) A typical example of the stimulus selection procedure used in the simulations. The brackets represent the set within which a stimulus can be randomly chosen. A not-detected stimulus results in an increase of the set while a detected stimulus results in a decrease of the set

The probability *p* of detecting a stimulus of amplitude *x* [mA] was modeled with a logistic psychophysical function:

1 $$ p\left(x;\;\alpha (t),\;\beta \right)={\left(1+ \exp\;\left(\beta \left(\alpha \left(\mathrm{t}\right)-\mathrm{x}\right)\right)\right)}^{-1}, $$

where *α*(*t*) and *β* were the threshold and slope parameter of the psychophysical function, respectively. Threshold drift over time was modeled as either a linear function *α*(*t*) = *α*_0_ + *θt*, with *θ* set to either 0 or 0.1 [(mA)/min], or as a saturating exponential function: $\alpha (t)={\alpha}_0+\left(1- \exp\;\left(-\frac{t}{\tau}\right)\right)$ , with the time-constant *τ* set to 1 or 3 [min]. The initial threshold *α*_0_ and slope *β* were set to 0.3 [mA] and 20 [1/(mA)], respectively. All simulated thresholds over time are presented in Fig. 2a.

Simulated responses to a given stimulus were classified as *detected* if *ε* < *p*(*x*), where ε is a random number drawn from a uniform distribution between 0 and 1, and as *not detected* otherwise. The random number generator was shuffled each time a new simulation was started to ensure uncorrelated realizations of simulated responses.

## Stimulus selection

For probing the modeled psychophysical functions by SRPs, stimulus amplitudes, *x*, were selected according to an adaptive probing procedure^1^ described in (Doll et al., 2014). The procedure started with a predefined set of 5 equidistant stimulus amplitudes between 0 and 0.3 mA, from which new stimulus amplitudes were randomly selected. All amplitudes in the set were increased by a step size of 0.1 mA after a not-detected stimulus and decreased with 0.1 mA after a detected stimulus (Fig. 2b).

In our experimental paradigm, the time between two consecutive stimuli depends on the corresponding response to the previous stimulus. Human subjects hold a response button in order for the equipment to start applying electrical stimuli. If the button is not released after a stimulus is applied, a *not-detected* stimulus is assumed. After a stimulus is *detected*, subjects are to release the button, and repress it again after about a second. This procedure results in a shorter interstimulus interval after a not-detected stimulus than after a detected stimulus. In the simulations described in this study, interstimulus intervals were set to 1.5 and 3.5 seconds after a not-detected stimulus and a detected stimulus, respectively.

## Threshold and slope estimation

Three estimation procedures were used to obtain threshold and slope estimates. The first and second estimation procedures were generalized linear regression models with a logit link function. The first procedure assumed a stationary threshold and slope throughout the experiments, whereas the second procedure assumed that time can have a linear effect on the detection probability. From here on, we refer to the first and second procedures as the static and relaxed procedures, respectively.

The static procedure was of the form:

2 $$ logit\;\left(\pi \right)={b}_0+{b}_1X, $$

where *π* is the estimated detection probability, *b*_0_ the intercept parameter and *b*_1_ the slope parameter for the stimulus amplitude X (i.e. x in Eq. 1).The threshold was defined as the amplitude resulting in a 0.5 detection probability:

3 $$ \alpha =X\;\left(\pi =.5\right)=-\left(\frac{b_0}{b_1}\right) $$

The relaxed procedure was of the form:

4 $$ logit\;\left(\pi \right)={b}_0+{b}_1X+{b}_2T, $$

where *b*_2_ is the slope parameter for the time T (i.e. t in Eq. 1). The threshold *α*(t) was calculated by:

5 $$ \alpha (t)=X\left(t\left|\pi =.5\right.\right)=-\left(\frac{b_0+t{b}_2}{b_1}\right) $$

Single threshold and slope estimates for the static and relaxed procedure were obtained by including all the available SRPs in each realization. The threshold estimate for the relaxed procedure were based on the detection probability at t = 5 minutes.

For threshold tracks, a shifting time window was used to obtain momentary estimates of the threshold and the slope using the regression model used for the relaxed procedure (i.e., Eq. 5 with t = the time at the center of the window). The window included 25 SRPs: 12 preceding SRPs, 12 upcoming SRPs, and the current SRP. This resulted in several threshold and slope estimates per realizations. Therefore, to obtain a single estimate, the momentary estimates were averaged resulting in a single threshold and slope estimate per realization.

## Analysis

Threshold and slope estimates were only included for analysis when the threshold estimate was between 0 and 5 mA and when the slope estimate was smaller than 50 [1/(mA)]. All other estimates were considered unrealistic and therefore were excluded from the dataset. Slope estimates were found to be skewed and were therefore natural log-transformed. To produce equally spaced estimates, threshold and slope tracks were linearly interpolated using a rate of 1 Hz to prevent under sampling.

## Human subject experiment

Recordings of two human subjects who show a non-stationary threshold were selected from a previous study (Doll et al., 2014). These were used to illustrate the different estimation procedures described earlier. Single cathodic square-wave electrical stimuli (with a pulse width of 525 μs) using a 5-needle electrode (Steenbergen et al., 2012) were presented to the subjects’ left forearm for 10 minutes. Single threshold estimates were obtained using both the static and relaxed procedures. Moreover, the threshold was tracked over time using the 25 most recent SRPs in the moving time window to obtain a momentary threshold. The relaxed procedure was used for threshold and slope estimation.

## Results

### Simulations

**Fig. 3:**
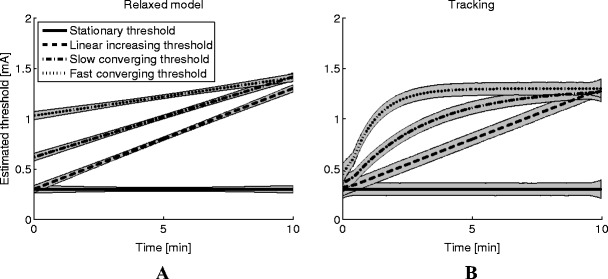
Mean and 95% confidence intervals of time-dependent estimated thresholds (i.e., for the relaxed (**a**) and tracking (**b**) procedures)

The mean and 95% confidence intervals of threshold estimates over time obtained by the relaxed procedure and the tracking procedure are presented in Fig. 3. The estimates are clearly dependent on time. The relaxed procedure approximates the nonlinear drift in the true threshold as linear drift, and, therefore results in estimations over time that do not follow the exponential form of the slow and fast converging thresholds. The tracking procedure, however, does follow the drift in threshold, regardless of the modeled drift.

**Fig. 4:**
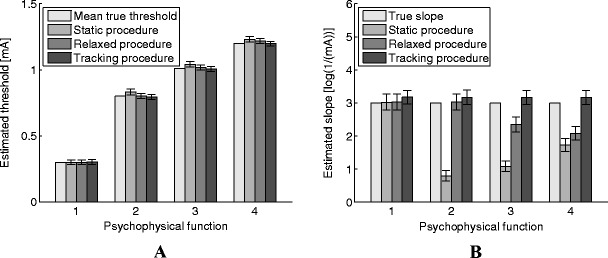
Mean and 95% confidence intervals of estimated thresholds (**a**) and natural log-transformed slopes (**b**) for the different psychophysical functions (1 indicates the constant threshold, 2 the linear increasing threshold, 3 the slow converging threshold, and 4 the fast converging threshold)

Figure 4a presents the mean true threshold and the mean and 95% confidence intervals of single threshold estimates obtained by all estimation procedures and all simulated psychophysical functions. Larger threshold estimates were obtained when the true threshold was increasing over time than when the threshold was kept constant. However, the estimates were similar to the mean true threshold for all estimation procedures.

Figure 4b presents the mean and 95% confidence intervals of single log-transformed slope estimates obtained by all estimation procedures and all simulated psychophysical functions. When the true psychophysical function remained stationary, slope estimates were similar to the true slope. When the static procedure was used to estimate slopes of non-stationary psychophysical functions, slopes were underestimated, resulting in more gradual functions than the true function. Slopes were underestimated only when the true threshold was described as a saturated exponential function and estimated using the relaxed procedure. Slope estimates remained similar to the log transformed true slope value when using the tracking method, regardless of the simulated psychophysical function.

### Human subject experiment

**Fig. 5:**
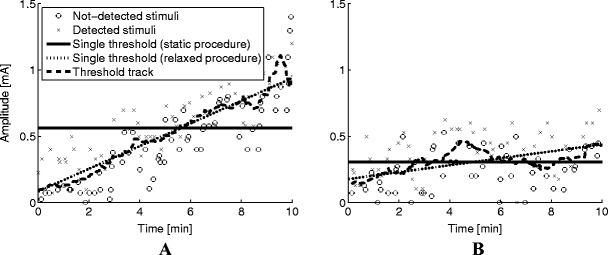
Human subject data of two subjects. Both graphs present stimuli and corresponding responses, where the crosses and circles indicate detected and not-detected stimuli, respectively. The solid black and dotted line represent a single estimated threshold using the static procedure (Eq. 2) and the relaxed procedures (Eq. 4), respectively. The dashed line represent the threshold track. The subject in (**a**) shows an increase in threshold over time, which could be assumed to be linear. However, the subject in (**b**) shows an effect of time on the threshold where linearity is debatable

Figure 5 presents the data of two human subjects where the stimulus response pairs and corresponding threshold estimates are plotted. For subject A, a threshold estimate of 0.56 mA was obtained when the static procedure was used. Using the relaxed procedure resulted in threshold estimates ranging between 0.09 and 0.95 mA from the start to the end of the experiment. Thresholds estimated by the tracking procedure resulted in similar estimates as the relaxed procedure. Estimating a single threshold for subject B, using the static procedure, resulted in an estimate of 0.31 mA. Using the relaxed procedure resulted in an estimate starting at 0.18 mA and ending at 0.44 mA at the end of the experiment. The threshold track estimated that the threshold increased from 0.18 to 0.45 mA from the start until approximately 5 minutes of the experiment. It then gradually decreased to 0.25 mA until 8 minutes and then continued to increase again to approximately 0.44 mA.

**Fig. 6:**
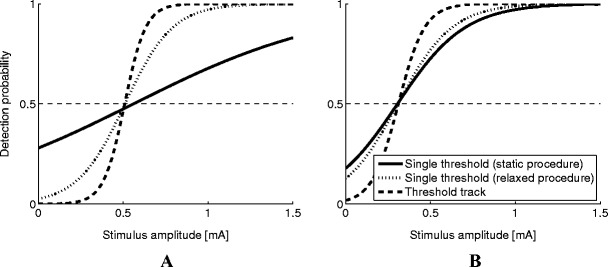
Estimated psychophysical functions using the different estimation procedures for the two human subjects

The psychophysical functions, obtained by estimating a single threshold and slope for each of the estimation procedures and each subject, are shown in Fig. 6. Estimated thresholds are similar for the three estimation procedures. However, estimates of the slope are not similar. The slope for subject A was estimated to be 0.52, 1.96, or 2.78 [log(1/(mA))] by using the static, relaxed, or tracking procedure, respectively. The slope for subject B was estimated to be 1.61, 1.84, or 2.56 [log(1/(mA))] by using the static, relaxed, or tracking procedure, respectively.

## Discussion

We used a Monte Carlo simulation approach to study the effect of non-stationary processes underlying the psychophysical function on the estimation of psychophysical thresholds and slopes. While the slope of the true psychophysical function was kept constant, the threshold was modeled as function of time. Estimates of the psychophysical threshold and slope were obtained from simulated stimulus response pairs, using three estimation procedures: static procedure, relaxed procedure, and threshold tracking procedure. In addition, we demonstrated the estimation procedures in two healthy human subjects for illustrative purposes.

When the processes underlying the psychophysical function resulted in a constant true threshold, estimates of the threshold obtained by the three estimation procedures were similar to the true threshold. Moreover, when the stationarity assumption was violated due to a drifting threshold, threshold estimates were similar to the mean true threshold value for all estimation procedures. However, it should be noted that approximately 240 SRPs were used for threshold estimation in this experiment. Given this number of SRPs, a high estimation precision is expected, which might be the reason why no relevant difference between the estimation procedures was found. Generally, the reliability of threshold estimates increases when more SRPs are included in the estimation process (Taylor, 1971). When fewer SRPs are available (e.g., fewer than 50), a difference between the results coming from the different estimation procedures might exist. Therefore, all three estimation procedures can be used to obtain threshold estimates whenever the threshold is the only parameter of interest in an experiment and a large number of SRPs are available.

When not only a threshold estimate is of interest, but also the reliability of stimulus detection by the subject, an estimate of the slope can be obtained as well. From the simulation results, it was found that slope estimates were similar whenever the threshold was constant over time. However, the static procedure returned biased slope estimates when the threshold was drifting over time. As mentioned in the introduction, the underestimation is due to the averaging of the psychophysical functions over time (Fig. 1). Accounting for a linear effect of time on the threshold by using the relaxed procedure improved the slope estimates (Fig. 4). However, even though estimates obtained by the relaxed procedure were better than the static procedure, they were still biased whenever the threshold was modeled as a nonlinear function. This bias was no longer visible when the tracking paradigm was used to obtain threshold and slope estimates. As the slope estimates obtained by the tracking procedure are similar or better than those obtained by the other two procedures, we recommend preferring the tracking procedure over the other procedures in experiments where estimates of the slope are required.

In addition to threshold and slope estimates, an estimate of the effect of time on the threshold can be obtained by the relaxed procedure and tracking procedure. The relaxed procedure assumes that the threshold drifts over time with a linear rate. Nonlinear threshold changes are approximated as linear function, resulting in estimation biases. This is not the case when the tracking procedure was used; the tracking procedure assumes piecewise linearity of the threshold resulting in a lower bias, even when the threshold drifts with a nonlinear rate. However, as shown in Fig. 3, the estimation confidence intervals are smallest when the relaxed procedure was used. This implies that the threshold estimation precision per time-point is higher when the relaxed procedure was used than when the tracking procedure was used. Therefore, if it is reasonable to state that the underlying process is either stationary or results in a linear drifting threshold, the relaxed procedure performs better than the tracking procedure. The tracking procedure could be used to obtain an indication on whether linearity could be assumed. For example, from the data obtained for subject A (Figs. 5a and 6a), it becomes immediately clear that the psychophysical curve obtained by the static procedure results in a poor fit but seems to be fit better by both the relaxed and tracking procedures. For subject B, however, it is not immediately clear what model to fit to the data. The threshold track shows a non-monotonously drifting threshold, suggesting that neither the static nor the relaxed procedure is appropriate. Therefore, in human subject experiments, the tracking procedure can be used as an indication for threshold behavior over time. If linearity seems reasonable, the relaxed procedure can be used to obtain precise threshold estimates, otherwise, the tracking procedure is recommended.

In this study, a threshold was modeled as either constant or monotonous increasing. However, in practice, non-monotonous varying thresholds may occur as well. For example, in pain experiments, a conditioning stimulus, such as immersing an extremity into painful cold water, might induce a temporary change in the threshold (Pud et al., 2009). Estimating a single threshold in these cases does not reflect any dynamic properties of the underlying processes. Therefore, a time-profile of the threshold could then be obtained by either estimating several single thresholds during the experiment or by tracking the threshold using the tracking paradigm.

Another limitation of the present study was that the slope of the psychophysical function was defined to have a constant value. In human subject experiments, however, the slope might show changes over time as well. Assuming a stationary slope, while the slope is non-stationary is likely to affect the estimation process. However, because we did not model a time-dependent slope, further studies are necessary to identify its effect on the estimation quality.

## Conclusions

We demonstrated that non-stationarities in processes underlying the psychophysical function resulting in threshold drift affect the estimation of thresholds and slopes. Slopes were underestimated, resulting in more gradual psychophysical functions than the true one. Accounting for linear effects of time on the threshold in the estimation process improves the slope estimates. However, slopes are still underestimated when the threshold drifts in a way that is not accounted for in the estimation model (i.e., nonlinear drift). Tracking the psychophysical function over time using a window shifting over time, and then averaging all estimates to obtain a single estimate, results in better threshold and slope estimates, regardless of the non-stationarity. We recommend using the tracking procedure in human psychophysical detection experiments to obtain estimates of the threshold and slope and to identify the mode of non-stationarity.

## Footnotes

1 In the cited reference this procedure was named “random staircase procedure”. However, we propose to change this into “adaptive probing procedure” to avoid confusion with the random staircase procedure described by e.g. Cornsweet (1962).
